# {2,7-Dieth­oxy-8-[(naphthalen-2-yl)carbon­yl]naphthalen-1-yl}(naphthalen-2-yl)methanone

**DOI:** 10.1107/S1600536813003577

**Published:** 2013-02-09

**Authors:** Takehiro Tsumuki, Akiko Okamoto, Hideaki Oike, Noriyuki Yonezawa

**Affiliations:** a Department of Organic and Polymer Materials Chemistry, Tokyo University of Agriculture & Technology, 2-24-16 Naka-machi, Koganei, Tokyo 184-8588, Japan

## Abstract

In the title compound, C_36_H_28_O_4_, the two 2-naphthoyl groups at the 1- and 8-positions of the central 2,7-dieth­oxy­naphthalene ring system are aligned almost anti­parallel and make a dihedral angle of 48.35 (5)°. The dihedral angles between the central 2,7-dieth­oxy­naphthalene ring system and the terminal naphthalene ring systems are 77.64 (4) and 73.73 (4)°. In the crystal, mol­ecules are linked into chains along the *a*-axis direction by dual C—H⋯O inter­actions between naphthoyl groups.

## Related literature

For electrophilic aroylation of naphthalene derivatives, see: Okamoto & Yonezawa (2009 ▶); Okamoto *et al.* (2011 ▶). For the structures of closely related compounds, see: Nakaema *et al.* (2008 ▶); Tsumuki *et al.* (2011 ▶); Sasagawa *et al.* (2012 ▶); Isogai *et al.* (2013 ▶); Yoshiwaka *et al.* (2013 ▶).
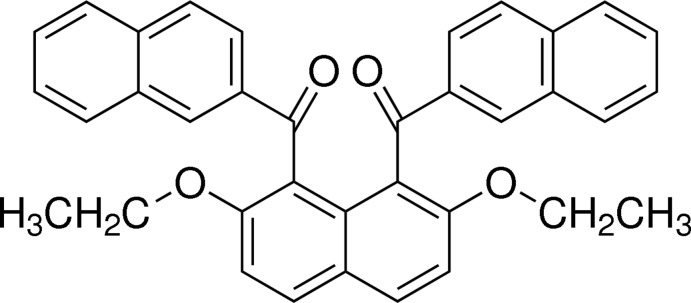


## Experimental

### 

#### Crystal data

C_36_H_28_O_4_
*M* *_r_* = 524.58
Monoclinic, 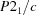

*a* = 7.86946 (14) Å
*b* = 27.1458 (5) Å
*c* = 12.8490 (2) Å
β = 102.267 (1)°
*V* = 2682.16 (8) Å^3^
*Z* = 4
Cu *K*α radiation
μ = 0.67 mm^−1^
*T* = 193 K
0.50 × 0.25 × 0.20 mm

#### Data collection

Rigaku R-AXIS RAPID diffractometer
Absorption correction: numerical (*NUMABS*; Higashi, 1999 ▶) *T*_min_ = 0.732, *T*_max_ = 0.878
41696 measured reflections
4914 independent reflections
3996 reflections with *I* > 2σ(*I*)
*R*_int_ = 0.029

#### Refinement

*R*[*F* ^2^ > 2σ(*F* ^2^)] = 0.036
*wR*(*F* ^2^) = 0.097
*S* = 1.09
4914 reflections
364 parameters
H-atom parameters constrained
Δρ_max_ = 0.20 e Å^−3^
Δρ_min_ = −0.15 e Å^−3^

### 

Data collection: *PROCESS-AUTO* (Rigaku, 1998 ▶); cell refinement: *PROCESS-AUTO*; data reduction: *CrystalStructure* (Rigaku, 2010 ▶); program(s) used to solve structure: *SIR2004* (Burla *et al.*, 2005 ▶); program(s) used to refine structure: *SHELXL97* (Sheldrick, 2008 ▶); molecular graphics: *ORTEPIII* (Burnett & Johnson, 1996 ▶); software used to prepare material for publication: *SHELXL97*.

## Supplementary Material

Crystal structure: contains datablock(s) I, global. DOI: 10.1107/S1600536813003577/rz5043sup1.cif

Structure factors: contains datablock(s) I. DOI: 10.1107/S1600536813003577/rz5043Isup2.hkl

Supplementary material file. DOI: 10.1107/S1600536813003577/rz5043Isup3.cml

Additional supplementary materials:  crystallographic information; 3D view; checkCIF report

## Figures and Tables

**Table 1:** Hydrogen-bond geometry (Å, °)

*D*—H⋯*A*	*D*—H	H⋯*A*	*D*⋯*A*	*D*—H⋯*A*
C21—H21⋯O3^i^	0.95	2.45	3.3958 (18)	173
C25—H25⋯O4^ii^	0.95	2.45	3.3996 (18)	176

Symmetry codes: (i) 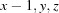; (ii) 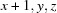.

**Fig. 1.**
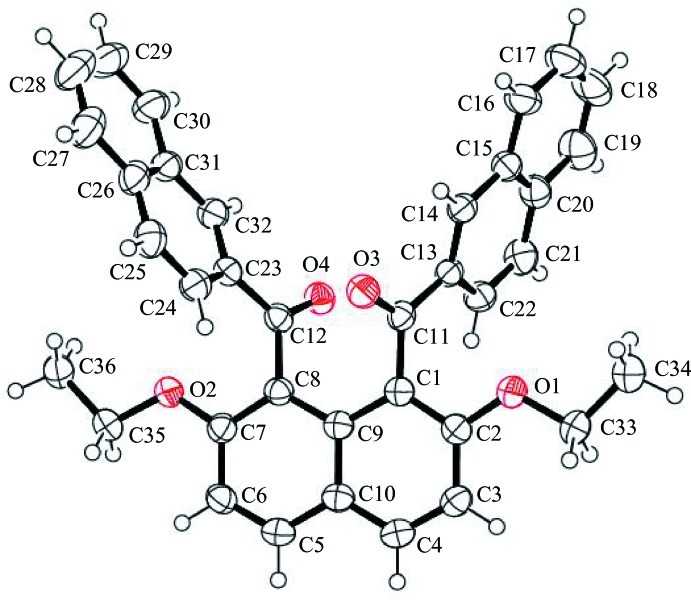
The molecular structure of the title molecule, with displacement ellipsoids drawn at the 50% probability level.

**Fig. 2.**
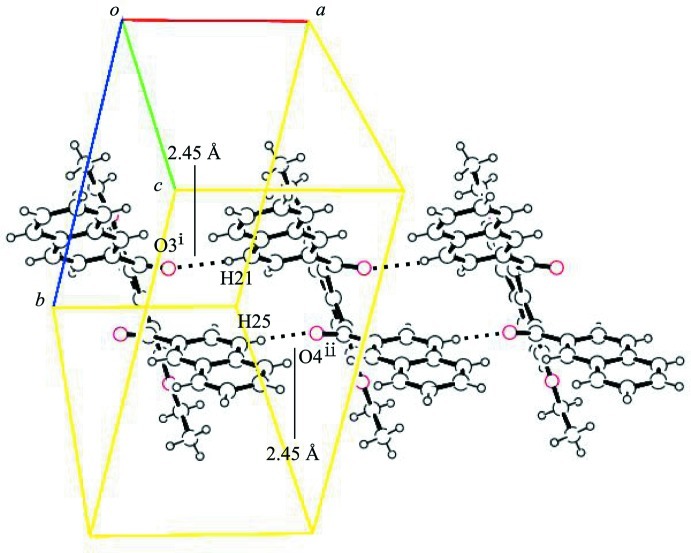
A partial view of the crystal packing of the title compound, showing the intermolecular C—H···O hydrogen bonds (see Table 1 for details; symmetry codes: (i) -1 + x, y, z; (ii) 1 + x, y, z).

## supplementary crystallographic information

### Comment

In the course of our study on selective electrophilic aromatic aroylation of the naphthalene ring core, 1,8-diaroylnaphthalene compounds have proved to be formed regioselectively by the aid of a suitable acidic mediator (Okamoto & Yonezawa, 2009, Okamoto *et al.*, 2011). Recently, we have reported the crystal structures of several 1,8-diaroylated naphthalene analogues exemplified by 1,8-dibenzoyl-2,7-dimethoxynaphthalene (Nakaema *et al.*, 2008), [2,7-dimethoxy-8-(2-naphthoyl)naphthalen-1-yl](naphthalen-2-yl)methanone (Tsumuki *et al.*, 2011), {2,7-dimethoxy-8-[4-(2-methylpropyl)benzoyl]naphthalen-1-yl}[4-(2-methylpropyl) phenyl]methanone (Sasagawa *et al.*, 2012), (8-benzoyl-2,7-diethoxynaphthalen-1-yl)(phenyl)methanone (Isogai *et al.*, 2013), and [8-(4-phenoxybenzoyl)-2,7-bis(propan-2-yloxy)naphthalen-1-yl](4-phenoxyphenyl) methanone (Yoshiwaka *et al.*, 2013). The most simple analogues in these compounds, 1,8-dibenzoyl-2,7-dimethoxynaphthalene (Nakaema *et al.*, 2008) and [2,7-dimethoxy-8-(2-naphthoyl)naphthalen-1-yl](naphthalen-2-yl)methanone (Tsumuki *et al.*, 2011), lie across a crystallographic 2-fold axis and the molecular packing are stabilized by C—H···O interactions and π···π interactions between the aroyl groups. As a part of our ongoing studies on the molecular structures of these kinds of homologous molecules, the X-ray crystal structure of the title compound is reported on herein.

The molecular structure of the title molecule is illustrated in Fig. 1. The two terminal naphthoyl groups are situated in an opposite direction and twisted away from the central 2,7-diethoxynaphthalene unit. The dihedral angles between the two naphthalene rings of the terminal naphthoyl groups (C13—C22 and C23—C32) is 48.35 (5)°. The dihedral angles between the terminal napthalene rings and the central naphthalene ring (C1—C10) are 77.64 (4) and 73.73 (4)°. The torsion angles between the carbonyl moieties and the central naphthalene ring are -52.63 (18)° [C9—C1—C11—O3] and -58.16 (17)° [C9—C8—C12—O4]. On the other hand, the carbonyl groups are slightly twisted away from the attached terminal naphthalene rings [torsion angles O3—C11—C13—C14 = -21.17 (19)° and O4—C12—C23—C32 = -14.10 (19)°]. In the crystal, the molecular packing of the title compound is mainly stabilized by two C—H···O interactions between the naphthoyl moieties leading to the formation of chains along the *a* axis (Table 1 and Fig. 2).

### Experimental

To a solution of 2-naphthoyl chloride (14.3 g, 75.0 mmol) and TiCl_4_ (42.7 g, 225 mmol) in CH_2_Cl_2_ (62.5 ml), 2,7-diethoxynaphthalene (5.4 g, 25.3 mmol) was added. The reaction mixture was stirred at r. t. for 24 h, then poured into ice-cold water (200 ml). The aqueous layer was extracted with CHCl_3_ (60 ml × 3). The combined organic extracts were washed with 2*M* aqueous NaOH (80 ml × 3) followed by washing with brine (80 ml × 3). The organic layer was dried over anhydrous MgSO_4_. The solvent was removed under reduced pressure to give a cake (yield 97%). The crude product was purified by recrystallization from chloroform/methanol (1:2 *v*/*v*) solution (isolated yield 70%). Furthermore, the isolated product was crystallized from chloroform to give single crystals suitable for X-ray analysis.

^1^H NMR δ (500 MHz, CDCl_3_): 0.86 (6*H*, t, *J* = 6.9 Hz), 3.96 (4*H*, q, *J* = 6.9), 7.21 (2*H*, d, *J* = 9.0 Hz), 7.39 (2*H*, t, *J* = 7.5 Hz), 7.47 (2*H*, t, *J* = 7.5 Hz), 7.69–7.93 (8*H*, m), 7.98 (2*H*, d, *J* = 9.0 Hz), 8.15 (2*H*, s) p.p.m.; ^13^C NMR δ (125 MHz, CDCl_3_): 14.36, 64.93, 112.35, 122.03, 124.89, 125.56, 125.89, 127.47, 127.54, 127.70, 129.59, 130.44, 130.74, 132.08, 132.41, 135.43, 136.47, 155.97, 197.17 p.p.m.; IR (KBr): 1658, 1623, 1608, 1510, 1470, 1275 cm^-1^; HRMS (*m/z*): [*M*+H]+ calcd. for C_36_H_29_O_4_, 525.2066; found, 525.2031.

### Refinement

All the H atoms were located in a difference Fourier map and were subsequently refined as riding atoms: C—H = 0.95 (aromatic), 0.98 (methyl) and 0.99 (methylene) Å, with *U*iso(H) = 1.2 *U*eq(C).

### Crystal data

**Table:**

C_36_H_28_O_4_	*F*(000) = 1104
*M**_r_* = 524.58	*D*_x_ = 1.299 Mg m^−^^3^
Monoclinic, *P*2_1_/*c*	Melting point = 493.0–494.5 K
Hall symbol: -P 2ybc	Cu *K*α radiation, λ = 1.54187 Å
*a* = 7.86946 (14) Å	Cell parameters from 33371 reflections
*b* = 27.1458 (5) Å	θ = 3.3–68.2°
*c* = 12.8490 (2) Å	µ = 0.67 mm^−^^1^
β = 102.267 (1)°	*T* = 193 K
*V* = 2682.16 (8) Å^3^	Block, colourless
*Z* = 4	0.50 × 0.25 × 0.20 mm

### Data collection

**Table:**

Rigaku R-AXIS RAPID diffractometer	4914 independent reflections
Radiation source: rotating anode	3996 reflections with *I* > 2σ(*I*)
Graphite monochromator	*R*_int_ = 0.029
Detector resolution: 10.000 pixels mm^-1^	θ_max_ = 68.2°, θ_min_ = 3.3°
ω scans	*h* = −9→9
Absorption correction: numerical (*NUMABS*; Higashi, 1999)	*k* = −31→32
*T*_min_ = 0.732, *T*_max_ = 0.878	*l* = −15→15
41696 measured reflections	

### Refinement

**Table:**

Refinement on *F*^2^	Secondary atom site location: difference Fourier map
Least-squares matrix: full	Hydrogen site location: inferred from neighbouring sites
*R*[*F*^2^ > 2σ(*F*^2^)] = 0.036	H-atom parameters constrained
*wR*(*F*^2^) = 0.097	*w* = 1/[σ^2^(*F*_o_^2^) + (0.0448*P*)^2^ + 0.4544*P*] where *P* = (*F*_o_^2^ + 2*F*_c_^2^)/3
*S* = 1.09	(Δ/σ)_max_ = 0.001
4914 reflections	Δρ_max_ = 0.20 e Å^−^^3^
364 parameters	Δρ_min_ = −0.15 e Å^−^^3^
0 restraints	Extinction correction: *SHELXL97* (Sheldrick, 2008), Fc^*^=kFc[1+0.001xFc^2^λ^3^/sin(2θ)]^-1/4^
Primary atom site location: structure-invariant direct methods	Extinction coefficient: 0.00121 (13)

### Special details

**Table:**

Geometry. All e.s.d.'s (except the e.s.d. in the dihedral angle between two l.s. planes) are estimated using the full covariance matrix. The cell e.s.d.'s are taken into account individually in the estimation of e.s.d.'s in distances, angles and torsion angles; correlations between e.s.d.'s in cell parameters are only used when they are defined by crystal symmetry. An approximate (isotropic) treatment of cell e.s.d.'s is used for estimating e.s.d.'s involving l.s. planes.
Refinement. Refinement of *F*^2^ against ALL reflections. The weighted *R*-factor *wR* and goodness of fit *S* are based on *F*^2^, conventional *R*-factors *R* are based on *F*, with *F* set to zero for negative *F*^2^. The threshold expression of *F*^2^ > σ(*F*^2^) is used only for calculating *R*-factors(gt) *etc*. and is not relevant to the choice of reflections for refinement. *R*-factors based on *F*^2^ are statistically about twice as large as those based on *F*, and *R*- factors based on ALL data will be even larger.

### Fractional atomic coordinates and isotropic or equivalent isotropic displacement parameters (Å^2^)

**Table:**

	*x*	*y*	*z*	*U*_iso_*/*U*_eq_	
O1	0.07897 (13)	0.25900 (3)	0.51894 (8)	0.0457 (3)	
O2	0.55755 (13)	0.32784 (3)	1.03877 (7)	0.0436 (2)	
O3	0.39273 (11)	0.34984 (3)	0.63817 (7)	0.0400 (2)	
O4	0.21986 (11)	0.37405 (3)	0.83968 (8)	0.0431 (2)	
C1	0.24047 (16)	0.28182 (5)	0.68544 (10)	0.0338 (3)	
C2	0.16047 (17)	0.24468 (5)	0.61849 (10)	0.0365 (3)	
C3	0.17099 (18)	0.19490 (5)	0.65104 (11)	0.0403 (3)	
H3	0.1137	0.1700	0.6047	0.048*	
C4	0.26404 (18)	0.18312 (5)	0.74950 (11)	0.0400 (3)	
H4	0.2736	0.1495	0.7705	0.048*	
C5	0.44227 (18)	0.20619 (5)	0.92342 (11)	0.0402 (3)	
H5	0.4541	0.1723	0.9422	0.048*	
C6	0.51793 (18)	0.24052 (5)	0.99566 (11)	0.0401 (3)	
H6	0.5833	0.2308	1.0634	0.048*	
C7	0.49772 (17)	0.29080 (5)	0.96834 (10)	0.0360 (3)	
C8	0.40829 (16)	0.30593 (5)	0.86876 (10)	0.0334 (3)	
C9	0.33228 (16)	0.26999 (5)	0.79099 (10)	0.0334 (3)	
C10	0.34690 (16)	0.21927 (5)	0.82158 (10)	0.0358 (3)	
C11	0.24972 (17)	0.33218 (5)	0.63750 (10)	0.0339 (3)	
C12	0.37086 (16)	0.36012 (5)	0.85329 (10)	0.0336 (3)	
C13	0.08776 (17)	0.35910 (5)	0.58868 (10)	0.0357 (3)	
C14	0.09312 (19)	0.39523 (5)	0.51522 (11)	0.0410 (3)	
H14	0.1997	0.4019	0.4944	0.049*	
C15	−0.0564 (2)	0.42290 (5)	0.46955 (11)	0.0443 (3)	
C16	−0.0535 (2)	0.46069 (6)	0.39398 (13)	0.0585 (4)	
H16	0.0507	0.4672	0.3703	0.070*	
C17	−0.1985 (3)	0.48786 (7)	0.35494 (14)	0.0710 (5)	
H17	−0.1945	0.5133	0.3047	0.085*	
C18	−0.3539 (3)	0.47841 (7)	0.38869 (14)	0.0735 (6)	
H18	−0.4544	0.4975	0.3608	0.088*	
C19	−0.3623 (2)	0.44224 (7)	0.46071 (13)	0.0624 (5)	
H19	−0.4684	0.4363	0.4826	0.075*	
C20	−0.21336 (19)	0.41325 (6)	0.50351 (12)	0.0473 (4)	
C21	−0.21620 (19)	0.37583 (6)	0.57927 (12)	0.0485 (4)	
H21	−0.3209	0.3692	0.6023	0.058*	
C22	−0.07049 (18)	0.34901 (5)	0.62000 (11)	0.0421 (3)	
H22	−0.0754	0.3234	0.6697	0.051*	
C23	0.51548 (16)	0.39574 (5)	0.85724 (10)	0.0331 (3)	
C24	0.68331 (17)	0.37994 (5)	0.84828 (11)	0.0406 (3)	
H24	0.7053	0.3458	0.8409	0.049*	
C25	0.81350 (19)	0.41323 (6)	0.85009 (12)	0.0483 (4)	
H25	0.9250	0.4021	0.8431	0.058*	
C26	0.78459 (18)	0.46415 (5)	0.86224 (11)	0.0442 (3)	
C27	0.9164 (2)	0.50030 (7)	0.86551 (15)	0.0625 (5)	
H27	1.0286	0.4907	0.8569	0.075*	
C28	0.8826 (3)	0.54880 (7)	0.88092 (15)	0.0691 (5)	
H28	0.9722	0.5725	0.8831	0.083*	
C29	0.7192 (3)	0.56425 (6)	0.89353 (14)	0.0619 (5)	
H29	0.6988	0.5981	0.9055	0.074*	
C30	0.5895 (2)	0.53092 (5)	0.88874 (12)	0.0506 (4)	
H30	0.4778	0.5418	0.8961	0.061*	
C31	0.61786 (18)	0.48013 (5)	0.87293 (10)	0.0396 (3)	
C32	0.48516 (17)	0.44475 (5)	0.86871 (10)	0.0367 (3)	
H32	0.3722	0.4553	0.8740	0.044*	
C33	−0.01793 (18)	0.22392 (5)	0.44723 (11)	0.0400 (3)	
H33A	−0.1017	0.2063	0.4814	0.048*	
H33B	0.0609	0.1995	0.4252	0.048*	
C34	−0.11200 (19)	0.25281 (5)	0.35288 (11)	0.0445 (3)	
H34A	−0.0272	0.2694	0.3190	0.053*	
H34B	−0.1868	0.2775	0.3764	0.053*	
H34C	−0.1835	0.2305	0.3016	0.053*	
C35	0.65820 (18)	0.31522 (5)	1.14207 (10)	0.0419 (3)	
H35A	0.7612	0.2956	1.1352	0.050*	
H35B	0.5874	0.2956	1.1821	0.050*	
C36	0.7138 (2)	0.36274 (6)	1.19899 (13)	0.0588 (4)	
H36A	0.7934	0.3558	1.2671	0.071*	
H36B	0.6113	0.3802	1.2121	0.071*	
H36C	0.7729	0.3833	1.1550	0.071*	

### Atomic displacement parameters (Å^2^)

**Table:**

	*U*^11^	*U*^22^	*U*^33^	*U*^12^	*U*^13^	*U*^23^
O1	0.0550 (6)	0.0357 (5)	0.0401 (5)	−0.0040 (4)	−0.0040 (4)	−0.0021 (4)
O2	0.0533 (6)	0.0400 (5)	0.0338 (5)	−0.0012 (4)	0.0010 (4)	0.0004 (4)
O3	0.0368 (5)	0.0424 (5)	0.0414 (5)	−0.0046 (4)	0.0097 (4)	0.0002 (4)
O4	0.0330 (5)	0.0413 (5)	0.0545 (6)	0.0036 (4)	0.0084 (4)	−0.0037 (4)
C1	0.0329 (7)	0.0317 (7)	0.0369 (7)	0.0008 (5)	0.0080 (5)	0.0001 (5)
C2	0.0353 (7)	0.0371 (7)	0.0366 (7)	0.0010 (6)	0.0064 (6)	0.0002 (6)
C3	0.0423 (8)	0.0327 (7)	0.0450 (8)	−0.0016 (6)	0.0073 (6)	−0.0052 (6)
C4	0.0444 (8)	0.0303 (7)	0.0464 (8)	0.0019 (6)	0.0121 (6)	0.0018 (6)
C5	0.0442 (8)	0.0353 (7)	0.0421 (7)	0.0036 (6)	0.0116 (6)	0.0070 (6)
C6	0.0423 (8)	0.0409 (8)	0.0364 (7)	0.0020 (6)	0.0070 (6)	0.0057 (6)
C7	0.0353 (7)	0.0365 (7)	0.0369 (7)	−0.0010 (5)	0.0093 (6)	−0.0004 (6)
C8	0.0314 (7)	0.0341 (7)	0.0359 (7)	0.0003 (5)	0.0096 (5)	0.0006 (5)
C9	0.0306 (7)	0.0333 (7)	0.0379 (7)	0.0016 (5)	0.0106 (5)	0.0007 (5)
C10	0.0363 (7)	0.0329 (7)	0.0403 (7)	0.0018 (5)	0.0129 (6)	0.0008 (6)
C11	0.0377 (7)	0.0332 (7)	0.0313 (6)	−0.0023 (6)	0.0080 (5)	−0.0038 (5)
C12	0.0345 (7)	0.0359 (7)	0.0299 (6)	0.0026 (5)	0.0059 (5)	−0.0019 (5)
C13	0.0388 (7)	0.0319 (7)	0.0354 (7)	−0.0014 (5)	0.0052 (6)	−0.0023 (5)
C14	0.0442 (8)	0.0379 (7)	0.0405 (7)	−0.0013 (6)	0.0081 (6)	−0.0002 (6)
C15	0.0536 (9)	0.0372 (8)	0.0383 (7)	0.0054 (6)	0.0014 (6)	−0.0027 (6)
C16	0.0757 (11)	0.0484 (9)	0.0467 (9)	0.0077 (8)	0.0025 (8)	0.0062 (7)
C17	0.1020 (16)	0.0568 (11)	0.0466 (10)	0.0234 (10)	−0.0011 (10)	0.0063 (8)
C18	0.0869 (14)	0.0723 (12)	0.0513 (10)	0.0402 (11)	−0.0081 (10)	−0.0061 (9)
C19	0.0580 (10)	0.0704 (11)	0.0524 (10)	0.0232 (9)	−0.0027 (8)	−0.0097 (9)
C20	0.0497 (9)	0.0462 (8)	0.0415 (8)	0.0089 (7)	−0.0003 (7)	−0.0087 (6)
C21	0.0388 (8)	0.0562 (9)	0.0499 (9)	0.0008 (7)	0.0079 (7)	−0.0064 (7)
C22	0.0397 (8)	0.0423 (8)	0.0442 (8)	−0.0018 (6)	0.0085 (6)	0.0011 (6)
C23	0.0343 (7)	0.0331 (7)	0.0309 (6)	0.0025 (5)	0.0050 (5)	0.0017 (5)
C24	0.0379 (7)	0.0387 (8)	0.0451 (8)	0.0051 (6)	0.0090 (6)	−0.0004 (6)
C25	0.0355 (8)	0.0557 (9)	0.0550 (9)	0.0011 (7)	0.0126 (7)	−0.0009 (7)
C26	0.0448 (8)	0.0470 (8)	0.0409 (8)	−0.0075 (6)	0.0096 (6)	0.0013 (6)
C27	0.0538 (10)	0.0681 (12)	0.0683 (11)	−0.0161 (9)	0.0188 (8)	0.0019 (9)
C28	0.0800 (13)	0.0545 (11)	0.0735 (12)	−0.0304 (10)	0.0180 (10)	0.0014 (9)
C29	0.0816 (13)	0.0412 (9)	0.0642 (11)	−0.0145 (8)	0.0184 (9)	0.0015 (8)
C30	0.0653 (10)	0.0363 (8)	0.0507 (9)	−0.0037 (7)	0.0133 (7)	0.0025 (7)
C31	0.0463 (8)	0.0384 (7)	0.0334 (7)	−0.0030 (6)	0.0067 (6)	0.0027 (6)
C32	0.0369 (7)	0.0365 (7)	0.0358 (7)	0.0027 (6)	0.0058 (6)	0.0023 (6)
C33	0.0406 (7)	0.0390 (7)	0.0402 (7)	−0.0054 (6)	0.0084 (6)	−0.0063 (6)
C34	0.0436 (8)	0.0497 (9)	0.0393 (8)	−0.0073 (6)	0.0067 (6)	−0.0009 (6)
C35	0.0422 (8)	0.0483 (8)	0.0336 (7)	0.0020 (6)	0.0046 (6)	0.0024 (6)
C36	0.0677 (11)	0.0549 (10)	0.0465 (9)	−0.0021 (8)	−0.0043 (8)	−0.0030 (7)

### Geometric parameters (Å, º)

**Table:**

O1—C2	1.3602 (16)	C19—C20	1.422 (2)
O1—C33	1.4278 (16)	C19—H19	0.9500
O2—C7	1.3676 (16)	C20—C21	1.410 (2)
O2—C35	1.4351 (16)	C21—C22	1.365 (2)
O3—C11	1.2217 (15)	C21—H21	0.9500
O4—C12	1.2237 (15)	C22—H22	0.9500
C1—C2	1.3867 (18)	C23—C32	1.3653 (18)
C1—C9	1.4310 (18)	C23—C24	1.4164 (18)
C1—C11	1.5075 (17)	C24—C25	1.363 (2)
C2—C3	1.4117 (19)	C24—H24	0.9500
C3—C4	1.3583 (19)	C25—C26	1.415 (2)
C3—H3	0.9500	C25—H25	0.9500
C4—C10	1.4103 (19)	C26—C31	1.416 (2)
C4—H4	0.9500	C26—C27	1.422 (2)
C5—C6	1.3600 (19)	C27—C28	1.366 (3)
C5—C10	1.4086 (19)	C27—H27	0.9500
C5—H5	0.9500	C28—C29	1.394 (3)
C6—C7	1.4099 (19)	C28—H28	0.9500
C6—H6	0.9500	C29—C30	1.356 (2)
C7—C8	1.3852 (18)	C29—H29	0.9500
C8—C9	1.4332 (18)	C30—C31	1.418 (2)
C8—C12	1.5049 (18)	C30—H30	0.9500
C9—C10	1.4300 (18)	C31—C32	1.4112 (19)
C11—C13	1.4874 (18)	C32—H32	0.9500
C12—C23	1.4860 (18)	C33—C34	1.5004 (19)
C13—C14	1.3680 (19)	C33—H33A	0.9900
C13—C22	1.4145 (19)	C33—H33B	0.9900
C14—C15	1.414 (2)	C34—H34A	0.9800
C14—H14	0.9500	C34—H34B	0.9800
C15—C16	1.416 (2)	C34—H34C	0.9800
C15—C20	1.419 (2)	C35—C36	1.501 (2)
C16—C17	1.361 (2)	C35—H35A	0.9900
C16—H16	0.9500	C35—H35B	0.9900
C17—C18	1.406 (3)	C36—H36A	0.9800
C17—H17	0.9500	C36—H36B	0.9800
C18—C19	1.360 (3)	C36—H36C	0.9800
C18—H18	0.9500		
			
C2—O1—C33	119.78 (10)	C15—C20—C19	118.60 (15)
C7—O2—C35	118.77 (10)	C22—C21—C20	120.90 (14)
C2—C1—C9	119.74 (12)	C22—C21—H21	119.6
C2—C1—C11	117.44 (11)	C20—C21—H21	119.6
C9—C1—C11	122.15 (11)	C21—C22—C13	120.41 (13)
O1—C2—C1	115.90 (11)	C21—C22—H22	119.8
O1—C2—C3	122.58 (12)	C13—C22—H22	119.8
C1—C2—C3	121.45 (12)	C32—C23—C24	119.41 (12)
C4—C3—C2	119.16 (13)	C32—C23—C12	119.19 (11)
C4—C3—H3	120.4	C24—C23—C12	121.39 (12)
C2—C3—H3	120.4	C25—C24—C23	120.57 (13)
C3—C4—C10	122.03 (13)	C25—C24—H24	119.7
C3—C4—H4	119.0	C23—C24—H24	119.7
C10—C4—H4	119.0	C24—C25—C26	120.79 (13)
C6—C5—C10	122.08 (13)	C24—C25—H25	119.6
C6—C5—H5	119.0	C26—C25—H25	119.6
C10—C5—H5	119.0	C25—C26—C31	118.94 (13)
C5—C6—C7	118.92 (13)	C25—C26—C27	122.96 (14)
C5—C6—H6	120.5	C31—C26—C27	118.09 (14)
C7—C6—H6	120.5	C28—C27—C26	120.42 (17)
O2—C7—C8	115.41 (11)	C28—C27—H27	119.8
O2—C7—C6	122.84 (12)	C26—C27—H27	119.8
C8—C7—C6	121.69 (12)	C27—C28—C29	121.30 (16)
C7—C8—C9	119.81 (12)	C27—C28—H28	119.4
C7—C8—C12	117.29 (11)	C29—C28—H28	119.4
C9—C8—C12	122.16 (11)	C30—C29—C28	119.88 (16)
C10—C9—C1	118.13 (12)	C30—C29—H29	120.1
C10—C9—C8	117.81 (12)	C28—C29—H29	120.1
C1—C9—C8	124.05 (12)	C29—C30—C31	121.01 (16)
C5—C10—C4	121.03 (12)	C29—C30—H30	119.5
C5—C10—C9	119.59 (12)	C31—C30—H30	119.5
C4—C10—C9	119.37 (12)	C32—C31—C26	118.77 (13)
O3—C11—C13	121.04 (11)	C32—C31—C30	121.95 (13)
O3—C11—C1	118.52 (11)	C26—C31—C30	119.28 (13)
C13—C11—C1	120.43 (11)	C23—C32—C31	121.50 (12)
O4—C12—C23	121.13 (12)	C23—C32—H32	119.3
O4—C12—C8	118.55 (11)	C31—C32—H32	119.3
C23—C12—C8	120.30 (11)	O1—C33—C34	106.08 (11)
C14—C13—C22	119.55 (13)	O1—C33—H33A	110.5
C14—C13—C11	119.60 (12)	C34—C33—H33A	110.5
C22—C13—C11	120.81 (12)	O1—C33—H33B	110.5
C13—C14—C15	121.49 (13)	C34—C33—H33B	110.5
C13—C14—H14	119.3	H33A—C33—H33B	108.7
C15—C14—H14	119.3	C33—C34—H34A	109.5
C14—C15—C16	122.46 (15)	C33—C34—H34B	109.5
C14—C15—C20	118.43 (13)	H34A—C34—H34B	109.5
C16—C15—C20	119.08 (14)	C33—C34—H34C	109.5
C17—C16—C15	120.76 (18)	H34A—C34—H34C	109.5
C17—C16—H16	119.6	H34B—C34—H34C	109.5
C15—C16—H16	119.6	O2—C35—C36	106.97 (12)
C16—C17—C18	120.22 (18)	O2—C35—H35A	110.3
C16—C17—H17	119.9	C36—C35—H35A	110.3
C18—C17—H17	119.9	O2—C35—H35B	110.3
C19—C18—C17	120.83 (17)	C36—C35—H35B	110.3
C19—C18—H18	119.6	H35A—C35—H35B	108.6
C17—C18—H18	119.6	C35—C36—H36A	109.5
C18—C19—C20	120.51 (18)	C35—C36—H36B	109.5
C18—C19—H19	119.7	H36A—C36—H36B	109.5
C20—C19—H19	119.7	C35—C36—H36C	109.5
C21—C20—C15	119.19 (13)	H36A—C36—H36C	109.5
C21—C20—C19	122.21 (15)	H36B—C36—H36C	109.5
			
C33—O1—C2—C1	−175.31 (11)	C22—C13—C14—C15	0.0 (2)
C33—O1—C2—C3	7.58 (19)	C11—C13—C14—C15	−177.80 (12)
C9—C1—C2—O1	−178.78 (11)	C13—C14—C15—C16	179.53 (14)
C11—C1—C2—O1	−7.94 (17)	C13—C14—C15—C20	1.5 (2)
C9—C1—C2—C3	−1.64 (19)	C14—C15—C16—C17	−177.36 (15)
C11—C1—C2—C3	169.20 (12)	C20—C15—C16—C17	0.7 (2)
O1—C2—C3—C4	175.83 (12)	C15—C16—C17—C18	−0.5 (3)
C1—C2—C3—C4	−1.1 (2)	C16—C17—C18—C19	0.1 (3)
C2—C3—C4—C10	1.7 (2)	C17—C18—C19—C20	0.1 (3)
C10—C5—C6—C7	1.1 (2)	C14—C15—C20—C21	−1.5 (2)
C35—O2—C7—C8	−177.25 (11)	C16—C15—C20—C21	−179.60 (14)
C35—O2—C7—C6	5.50 (18)	C14—C15—C20—C19	177.69 (13)
C5—C6—C7—O2	174.74 (12)	C16—C15—C20—C19	−0.4 (2)
C5—C6—C7—C8	−2.3 (2)	C18—C19—C20—C21	179.21 (16)
O2—C7—C8—C9	−176.70 (11)	C18—C19—C20—C15	0.0 (2)
C6—C7—C8—C9	0.58 (19)	C15—C20—C21—C22	0.0 (2)
O2—C7—C8—C12	−6.34 (16)	C19—C20—C21—C22	−179.14 (14)
C6—C7—C8—C12	170.94 (12)	C20—C21—C22—C13	1.5 (2)
C2—C1—C9—C10	3.69 (17)	C14—C13—C22—C21	−1.5 (2)
C11—C1—C9—C10	−166.70 (11)	C11—C13—C22—C21	176.23 (12)
C2—C1—C9—C8	−175.25 (12)	O4—C12—C23—C32	14.09 (18)
C11—C1—C9—C8	14.35 (18)	C8—C12—C23—C32	−164.40 (12)
C7—C8—C9—C10	2.31 (17)	O4—C12—C23—C24	−165.25 (12)
C12—C8—C9—C10	−167.56 (11)	C8—C12—C23—C24	16.25 (18)
C7—C8—C9—C1	−178.74 (11)	C32—C23—C24—C25	−0.6 (2)
C12—C8—C9—C1	11.38 (19)	C12—C23—C24—C25	178.77 (13)
C6—C5—C10—C4	−177.56 (13)	C23—C24—C25—C26	0.8 (2)
C6—C5—C10—C9	1.8 (2)	C24—C25—C26—C31	0.2 (2)
C3—C4—C10—C5	179.80 (12)	C24—C25—C26—C27	179.46 (15)
C3—C4—C10—C9	0.4 (2)	C25—C26—C27—C28	−177.82 (16)
C1—C9—C10—C5	177.50 (11)	C31—C26—C27—C28	1.4 (2)
C8—C9—C10—C5	−3.49 (18)	C26—C27—C28—C29	−0.2 (3)
C1—C9—C10—C4	−3.10 (18)	C27—C28—C29—C30	−1.1 (3)
C8—C9—C10—C4	175.91 (11)	C28—C29—C30—C31	1.1 (3)
C2—C1—C11—O3	−117.99 (13)	C25—C26—C31—C32	−1.4 (2)
C9—C1—C11—O3	52.62 (17)	C27—C26—C31—C32	179.34 (14)
C2—C1—C11—C13	60.82 (16)	C25—C26—C31—C30	177.88 (14)
C9—C1—C11—C13	−128.58 (13)	C27—C26—C31—C30	−1.4 (2)
C7—C8—C12—O4	−111.96 (14)	C29—C30—C31—C32	179.38 (14)
C9—C8—C12—O4	58.15 (17)	C29—C30—C31—C26	0.1 (2)
C7—C8—C12—C23	66.57 (15)	C24—C23—C32—C31	−0.64 (19)
C9—C8—C12—C23	−123.31 (13)	C12—C23—C32—C31	180.00 (12)
O3—C11—C13—C14	21.18 (19)	C26—C31—C32—C23	1.62 (19)
C1—C11—C13—C14	−157.60 (12)	C30—C31—C32—C23	−177.63 (13)
O3—C11—C13—C22	−156.59 (13)	C2—O1—C33—C34	171.47 (11)
C1—C11—C13—C22	24.64 (18)	C7—O2—C35—C36	176.89 (12)

### Hydrogen-bond geometry (Å, º)

**Table:**

*D*—H···*A*	*D*—H	H···*A*	*D*···*A*	*D*—H···*A*
C21—H21···O3^i^	0.95	2.45	3.3958 (18)	173
C25—H25···O4^ii^	0.95	2.45	3.3996 (18)	176

Symmetry codes: (i) *x*−1, *y*, *z*; (ii) *x*+1, *y*, *z*.
